# A 1-Week Comprehensive Foam Rolling Intervention Program Can Improve Knee Pain but Not Muscle Function and Range of Motion in Patients with Total Knee Arthroplasty

**DOI:** 10.3390/ijerph20043351

**Published:** 2023-02-14

**Authors:** Masanobu Yokochi, Masatoshi Nakamura, Ayaka Iwata, Ryota Kaneko, Shiho Watanabe, Andreas Konrad, Noboru Yamada

**Affiliations:** 1 Department of Rehabilitation, Takeda General Hospital, 3-27 Yamagamachi, Aizuwakamatsu 965-8585, Fukushima, Japan; 2 Department of Rehabilitation, Fukushima Medical University, 1 Hikarigaoka, Fukushima City 960-1295, Fukushima, Japan; 3 Faculty of Rehabilitation Sciences, Nishi Kyushu University, 4490-9 Ozaki, Kanzaki 842-8585, Saga, Japan; 4 Institute of Human Movement Science, Sport and Health, Graz University, Mozartgasse 14, 8010 Graz, Austria; 5 Department of Orthopaedic Surgery, Takeda General Hospital, 3-27 Yamagamachi, Aizuwakamatsu 965-8585, Fukushima, Japan

**Keywords:** isometric contraction torque, walking speed, flexibility, foam rolling, knee arthroplasty, pain

## Abstract

We investigate the effect of a 1-week comprehensive foam rolling (FR) intervention program on knee pain, range of motion (ROM), and muscle function in patients with TKA.Thirty patients with TKA were randomly allocated to FR (n = 15) or control (n = 15) groups. The control group received only regular physical therapy. Patients in the FR group performed the FR intervention in addition to their regular physical therapy twice daily from postoperative weeks two to three (60 s × 3 repetitions × 2 times/day × 6 days: total = 2160 s). Pain score, knee flexion and extension ROM, muscle strength, walking function, and balance function were measured before and after the FR intervention. From the second to third postoperative weeks, there were significant improvements in all variables, and the reduction in pain score at stretching was significantly greater in the FR group (−26.0 ± 1.4; *p* < 0.05) than in the control group (−12.5 ± 1.9). However, there was no significant difference in changes in the other variables except for the pain score at stretching between FR and control groups. A 1-week comprehensive FR intervention program in patients with TKA could reduce pain scores at stretching without a synergistic effect on physical function, i.e., walking speed, balance function, and muscle strength of the knee extensors muscles.

## 1. Introduction

Total knee arthroplasty (TKA) can reduce pain and improve the knee joint’s range of motion (ROM) in patients with end-stage knee osteoarthritis (OA). However, previous studies have shown that TKA induces limited knee joint ROM and decreases motor functions, such as walking speed. It is also well-known that pain in the early postoperative period is associated with improved knee joint ROM, pain, and walking speed three months after TKA. Specifically, previous studies have shown a significantly slower walking speed and a significant impairment in the knee joint flexion angle in the early stance phase and knee joint extension angle in the middle stance phase in patients with TKA as compared with healthy subjects [1,2]. A previous study pointed out that these changes in walking mechanics could be associated with decreased functional performance and progression of contralateral knee OA [3]. In addition, the limitation in the ROM of the knee joint due to increased stiffness can persist after TKA. Previous studies have reported that knee stiffness occurs in 8–12% of TKA patients [4,5,6]. Witvrouw et al. reported that the limitation in knee flexion ROM resulting from increased stiffness after TKA surgery interferes with patients’ activities of daily living and causes increased chronic pain and decreased functional scores [7]. In our clinical practice, we have experienced many cases in which the patient cannot perform knee joint ROM exercises (i.e., stretching exercises) due to knee pain. The remaining stiffness could lead to limited knee joint ROM in TKA patients. A decrease in knee joint ROM might impair the patient’s daily living abilities and narrow their daily living activities. Thus, improving knee ROM can contribute to improved walking and activities of daily living in patients with TKA.

Recently, foam rolling (FR) has attracted attention as a method for relieving pain and improving ROM in the sports field. It can be assumed that FR has a favorable effect in patients with TKA since no knee flexion is required, as is in stretching. Nakamura et al. showed that a single FR intervention could decrease muscle soreness and increase ROM in eccentrically induced damaged muscles in healthy participants [8]. In addition, previous studies have reported an increase in pain thresholds and ROM for the gastrocnemius muscle after long-term FR intervention [9,10,11]. Moreover, FR interventions were shown to effectively improve ROM in patients with hip OA [12] and fibromyalgia [13]. There are various possible mechanisms for the pain-relieving effects of FR intervention, such as the relaxing effect it produces. Massaging the muscles increases parasympathetic nerve activity, reduces anxiety, and improves mood [14]. Pressure stimulation of the muscles with FR intervention can be expected to have a relaxation effect similar to that of massage and might reduce pain. Therefore, FR intervention could effectively decrease knee pain and increase knee joint ROM in patients with TKA.

However, the effect of an FR intervention program on patients with TKA remains unclear. Regarding balance dysfunction, a previous study showed that 3% of post-TKA patients fall during hospitalization [15]. Additionally, Kampitak et al. investigated the association between pain and TUG after TKA surgery and reported that TUG was faster with less pain [16]; if FR intervention can reduce pain, it may lead to improved balance function. Thus, in this study, we aimed to investigate whether a 1-week FR intervention program could effectively improve pain, knee ROM, walking speed, and balance functions in patients with TKA from the second to the third postoperative week. Based on previous studies [12,13], a 1-week FR intervention program could effectively improve pain, knee ROM, and motor function in TKA patients. In particular, unlike massage intervention, FR intervention has the advantage of being a self-care intervention. Thus, if an FR intervention effectively improves pain, knee joint ROM, walking speed, and balance function in patients with postoperative TKA, it can be applied as a new rehabilitation method.

## 2. Materials and Methods

### 2.1. Experimental Design

We used a randomized, repeated-measures experimental design to compare the effects of a 1-week FR intervention on patients with TKA. The FR intervention was performed from the second to third postoperative weeks (1 week). The FR intervention program consisted of 60s × 3 repetitions × 2 times/day × 6 days (for a total of 2160 s/week) performed on the knee extensors muscles by a physical therapist. Outcome variables were measured before (PRE) and after (POST) the FR intervention. We assessed knee flexion and extension ROM, pain (i.e., using a visual analog scale [VAS]), 10-m walk speed, timed up-and-go test (TUG), one-leg standing time, and maximal voluntary isometric contraction (MVIC) torques of the knee extensors.

### 2.2. Participants

**Figure 1:**
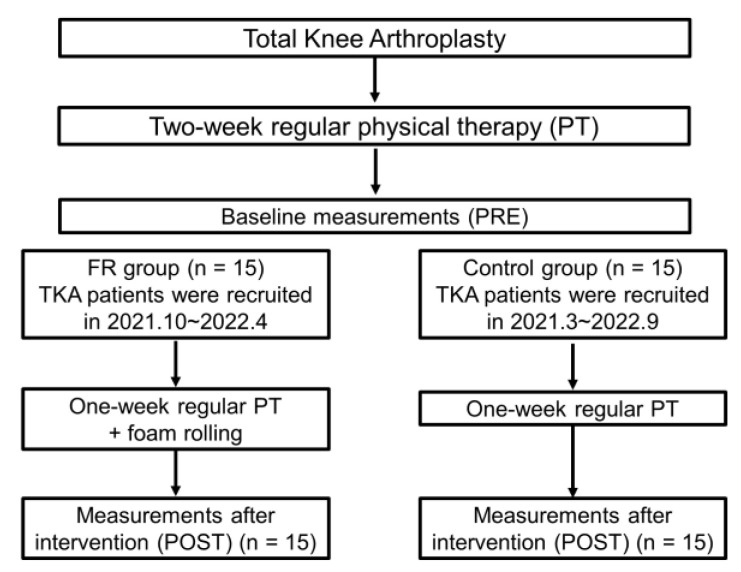
Flow chart of the study protocol.

The study population consisted of 30 patients admitted to our hospital who underwent TKA for knee OA (Figure 1). Participants were randomly assigned to the FR group (n = 15) or the control group (n = 15). The control group was hospitalized from March 2021 to September 2022 and received only regular physical therapy after the TKA procedure; the FR group was hospitalized from October 2021 to April 2022 and received the FR intervention program described below in addition to regular physical therapy for one week beginning the second week after surgery. The requested sample size using a split-plot analysis of variance (ANOVA) (effect size = 0.40, alpha error = 0.05, power = 0.80) using G*power 3.1 software (Heinrich Heine University, Düsseldorf, Germany) was more than 14 participants for each group.

The mean age of the FR group was 75.9 ± 6.0 years (range: 61–83 years), and the mean body mass index (BMI) was 26.8 ± 2.9 kg/m^2^. Thirteen participants were female (86.7%); 10 patients had left knee arthroplasty, whereas five patients had right knee arthroplasty. The mean age of the control group was 72.7 ± 7.9 years (range: 54–83 years), and the mean BMI was 27.8 ± 5.6 kg/m^2^. Thirteen participants were female (86.7%); eight patients received left knee arthroplasty, whereas seven patients underwent right knee arthroplasty. There were no significant differences in age or BMI between the two groups. All patients underwent the medial parapatellar approach. Patients were excluded if they could not provide consent for the study or if they had rheumatoid arthritis in their current medical history. In this study, the subject takes analgesic medication according to the degree of pain and the doctor’s order. All 30 participants completed the study without dropping out. An ethical review was conducted after obtaining approval from the Ethical Review Committee of our hospital (approval No. R3-298). The study was registered with the University Hospital Medical Information Network Clinical TrialsRegistry (UMIN000050279; 9 February 2023).

### 2.3. Regular Physical Therapy Intervention

Regular physical therapy was started on the day after surgery. All participants performed wheelchair practice, ROM exercises, strength training, and walking training according to their pain and general conditions. Our TKA protocol is to perform walking exercises with a walker one week after surgery, with a goal of 90° knee flexion. Two weeks after surgery, patients practice walking with a T-cane with a goal of 100° knee flexion. Three weeks after surgery, the patient will practice climbing up and down steps to be discharged from the hospital. Physical therapy interventions were conducted for 40 min each in the morning and afternoon, for a total of 80 min.

### 2.4. FR Exercise

**Figure 2:**
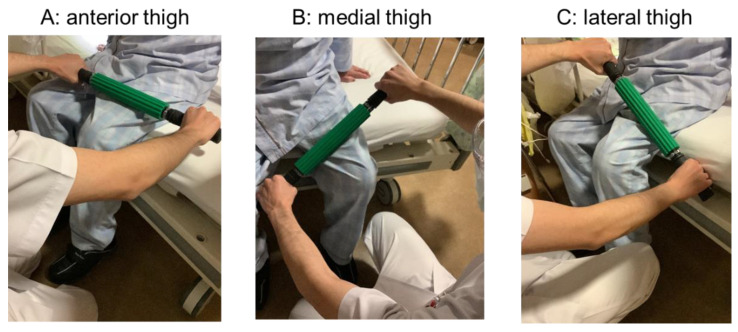
Foam rolling intervention for anterior thigh, medial thigh, and lateral thigh.

Our TKA protocol performs wound extraction in the second postoperative week. After the physical therapist confirmed there were no problems with the wound, a roller massager (TheraBand, Akron, OH, USA) was used in the second postoperative week after the extraction. FR was performed for 60 s each in the order of anterior thigh, medial thigh, and lateral thigh without a break, starting from a site 5 cm above the nearest edge of the surgical wound toward the hip joint, in accordance with a previous study [17] (Figure 2). The patients performed the exercises twice daily, in the morning and the afternoon, from the second to the third postoperative week (60s × 3 repetitions × 2 times/day × 6 days, for a total of 2160s). Another advantage of this study is that pain was reduced in a short period of time during the hospital stay, one week from the second to the third postoperative week, and no patients dropped out of the study due to increased pain or other reasons during the intervention.

### 2.5. Knee Flexion and Extension ROM Measurement

The physical therapist measured knee flexion and extension ROM using a goniometer [18]. Two physiotherapists with at least one year of experience performed the measurements, and the results were confirmed by the two physiotherapists.

The participant was laid in a supine position, and passive knee flexion and extension ROM that could be tolerated was measured. Measurements were performed on the bed. In this study, we were not able to blind the investigators to the intervention information. We have calculated the intraclass correlation coefficient (ICC) for knee flexion and extension ROM, and we confirmed the high reliabilities for knee extension and flexion ROM (ICC = 1.00 and 0.92, respectively).

### 2.6. Pain Measurement

Before FR intervention was performed in the second postoperative week, the degree of pain was measured using the VAS during knee joint ROM measurement. The VAS allows patients to estimate knee pain by marking an X on a 100-mm line (0 mm for no pain, 100 mm for worst possible pain). The pain was defined as the pain at the maximum ROM the patient could tolerate. The VAS has the highest–retest reliability and has been shown to correlate with other tests of pain intensity [19]. Also, we confirmed high reliability for measurement (ICC = 0.997).

### 2.7. Walking Measurements

In the FR group, measurements were obtained before FR at two and three weeks postoperatively. Walking speed was measured using a stopwatch at a comfortable walking speed of 10 m. A 3-m spare path was set up at the start and end of each measurement line, and measurements were taken twice. For all variables, the average of two measurements was used for the statistical analysis. ICC (1, 2) were 0.979 in two weeks and 0.975 in three weeks postoperatively.

### 2.8. Balance Measurements

Balance was measured using the TUG [20] and the one-leg standing test, and measurements were obtained twice. In the one-leg standing posture, the time holding the posture without touching the support was measured on the examiner’s cue. For all variables, the average of two measurements was used for the statistical analysis. ICC (1, 2) for TUG were 0.963 in two weeks and 0.965 in three weeks postoperatively, and ICC (1, 2) for the one-leg standing test were 0.881 in two weeks and 0.837 in three weeks postoperatively.

### 2.9. Muscle Strength Measurement

We measured isometric knee extensor muscle strength using a handheld dynamometer (Micro FET 2; Nihon Medix, Chiba, Japan). Muscle strength measurements via HHD were performed by the same well-trained physical therapist with more than ten years of experience. The method was based on a previous study [21] in which physical therapists measured twice, and a 60s break was allowed between each measurement. The average value was used, and the mean value was divided by body weight for the statistical analysis. ICC (1, 2) for TUG were 0.958 in two weeks and 0.96 in three weeks postoperatively.

### 2.10. Statistical Analysis

SPSS (version 28.0; IBM Corp., Armonk, NY, USA) was used for statistical analyses. The distribution of the data was assessed using the Shapiro–Wilk test, and we confirmed that the data followed a normal distribution. To verify the consistency of baseline values (i.e., values in the second postoperative week), the PRE values were tested between the FR and control groups using an unpaired *t*-test. A split-plot analysis of variance (ANOVA) (time [PRE vs. POST] × groups [FR group vs. control group]) was used to identify the interactions and main effects. If the interaction effect was significant, we conducted a post hoc analysis using paired *t*-tests on each group to determine the difference between PRE and POST values. The significance level was set at 5%. All results are shown as mean ± SD.

## 3. Results

Table 1 shows the changes in outcome variables, and there were no significant differences in PRE values between the FR and control groups. Split-plot ANOVAs showed no significant interaction effects in knee flexion ROM (F = 0.017, *p* = 0.898), knee extension ROM (F = 0.84, *p* = 0.367), pain score at rest (F = 0.389, *p* = 0.554), walk speed (F = 0.069, *p* = 0.794), TUG (F = 1.04, *p* = 0.316), one-leg standing time (F = 0.952, *p* = 0.338), and MVIC (F = 0.073, *p* = 0.788), but the main effects are in these variables. Conversely, a split-plot ANOVA showed a significant interaction effect in pain scores at stretching (F = 5.14, *p* = 0.031), and a post hoc test revealed significant (*p* < 0.01) decreases in pain scores at stretching in both the FR and control groups. We calculated the changes from PRE to POST in both the FR and control groups, and the change in the FR group (−26.0 ± 1.4) was significantly (*p* < 0.05) greater than in the control group (−12.5 ± 1.9).

## 4. Discussion

In this study, we investigated the effects of a 1-week comprehensive FR intervention on pain, knee joint ROM, isometric knee extensor muscle strength, walking speed, and balance function from the second to the third postoperative week in patients with TKA. The results showed that adding one week of FR intervention to regular physical therapy significantly improved pain scores at stretching, but there was no synergistic effect on the other parameters.

In general, knee pain is known to occur after TKA, and Seo et al. reported that 60% of patients who underwent TKA experienced severe postoperative knee pain, whereas 30% experienced moderate pain [22]. Also, the previous study investigated the association between pain on postoperative days one and five and motor function at three months, and the results showed that the less pain in the early postoperative period, the better the motor function at three months postoperatively [23]. It is also well-known that pain in the early postoperative period is associated with improved knee joint ROM, pain, and walking speed three months after TKA. Therefore, improving physiotherapeutic interventions to decrease pain in the early postoperative period is necessary to control for improving knee joint ROM and walking functions [23]. The results of this study showed that one week of FR intervention on the anterior thigh from the second postoperative week resulted in a significant (*p* = 0.031) reduction in pain score at stretching (−26.0 ± 1.4) as compared with the control group (−12.5 ± 1.9). A previous study reported that minimal clinically important differences in pain improvement following TKA were 22.6 mm on the VAS [24]. The present study observed an improvement of 26 mm in the VAS in the FR group, which is considered a clinically significant change. Ikutomo et al. reported that a 12-week FR intervention improved hip pain, physical function, and QOL in patients with hip OA, as compared with the control group [12]. In addition, an FR intervention program could reduce pain in patients with fibromyalgia [25,26]. The results of our study were consistent with these previous findings [12,25,26]. Although the mechanism by which the FR intervention improved the pain score in this study is unknown, previous studies reported that an FR intervention increases the pain pressure threshold and pain tolerance (stretch tolerance) [9,11,24]. A systematic review suggests that rolling muscle and skin stimulation may increase temperature, and pressure stimulation of tissues is associated with increased pain thresholds [25]. In addition, the skin and fascia have been found to be highly innervated by sensory neurons, including Ruffini and Pacinian receptors, which may have the ability to inhibit sympathetic activity and induce muscle relaxation. Ruffini receptors, in particular, are sensitive to tangential force and lateral stretch, and it has been hypothesized that stimulation of these receptors through FR may improve pain and flexibility. Therefore, an FR intervention could be an effective physical therapy tool for pain relief in the early postoperative period after TKA.

On the other hand, we considered increasing knee joint ROM through FR intervention on the anterior thigh muscles, specifically the knee joint extensors; however, there was no significant interaction between knee flexion ROM in the FR and control groups. Kiyono et al. reported a significant increase in ROM after five weeks of an FR intervention [10]. In addition, a meta-analysis of the long-term intervention effects of FR intervention programs reported an increase in ROM with FR training of four weeks or longer [9]. Therefore, an FR intervention program of more than four weeks’ duration may be needed to effectively increase ROM. This study included only a 1-week FR intervention from the second to the third postoperative week; thus, the short duration of intervention in this study may have contributed to the lack of improvement in ROM and motor function, as the FR intervention was only administered for one week from the second to the third postoperative week.

Both the FR and Control (regular physical therapy only) groups significantly improved 10-m walking speed, TUG time, time to stand on one leg, and isometric knee extensor muscle strength from postoperative weeks two to three. However, there were no significant differences in the changes in these variables between the FR and control groups. A meta-analysis of the effects of the FR intervention on motor function in young subjects reported no change in performance after several weeks of FR intervention training programs [27]. On the other hand, in a previous study of patients with hip OA, the FR intervention was shown to improve walking ability, stair-climbing ability, activities of daily living, and quality of life. Although the FR intervention was performed for at least ten min per day for 12 weeks in this previous study [12], this study included a 1-week FR intervention from the second to the third postoperative week because FR intervention was available at this hospital during this period. Our results showed that pain was reduced from the second to the third postoperative week, and no patients dropped out of the study due to increased pain or other reasons during the intervention. Therefore, FR intervention may be effective in relieving pain during this period. While physical therapists administered the intervention in this study, patients may be able to alleviate pain through self-administration of the FR intervention. These results may provide useful information for postoperative pain management. Previous research has suggested that extending the intervention period may result in improvements in knee flexion ROM and motor function in TKA patients. Thus, the effect of a longer FR intervention program on the physical function of patients with postoperative TKA and the change in ROM should be investigated.

Stretching is a typical postoperative ROM exercise for TKA patients but stretching may also put stress on the wound. Conversely, FR intervention is an approach that can reduce pain by increasing tissue temperature and stimulating proprioceptors without putting stress on the wound. Additionally, FR intervention can approach the quadriceps muscle without putting stress on the wound. Therefore, FR intervention for the quadriceps muscle is used in clinical practice and, as a result, early postoperative knee pain can be suppressed. Early postoperative pain after TKA has been linked to persistent knee pain and reduced walking speed three months post-surgery, suggesting the importance of effective early pain control. Previous research has also found that the severity of pain on postoperative days one and five is associated with improved motor function at three months post-surgery [23]. FR intervention can be performed by patients and is therefore considered an effective tool for self-care during future rehabilitation. The FR intervention program may improve future knee pain and walking function by controlling knee pain in the early postoperative period after TKA. Thus, conducting a long-term follow-up study is necessary in the future.

## 5. Limitation

There were some limitations in this study. We did not investigate FR intensity. Also, FR intervention is typically not self-administered by patients, and this study has not been blinded. Future research should explore the effectiveness of self-administered FR intervention. In addition, all subjects were taking pain medication during the study period, but the time and dose after using the analgesic medication may vary among participants. Therefore, it is necessary to examine the time and dose after using the analgesic medication under uniform conditions in the future.

## 6. Conclusions

The results showed that, as compared with the control group, the FR intervention program significantly improved knee pain at stretching (knee flexion), but there was no synergistic effect on the other parameters.

## Figures and Tables

**Table 1:** Changes in outcome variables before (PRE) and after a 1-week comprehensive foam rolling (FR) program (POST).

	FR Group			Control Group		
	PRE	POST	Δ Change	PRE	POST	Δ Change
Knee flexion ROM (°)	97.0 ± 10.5	106.3 ± 9.5 *	9.3 ± 4.7	102.0 ± 10.8	111.0 ± 7.1 *	9.0 ± 4.5
	d=	0.96	*p* < 0.01	d=	1.04	*p* < 0.01
Knee extension ROM (°)	−4.0 ± 3.9	−3.0 ± 3.7 *	1.0 ± 0.5	−7.3 ± 5.6	−5.3 ± 4.0 *	2.0 ± 1
	d=	0.27	*p* < 0.01	d=	0.43	*p* < 0.01
Pain score at rest (mm)	9.9 ± 11.7	1.3 ± 3.0 *	−8.6 ± 4.3	17.1 ± 20.2	5.3 ± 13.6 *	−11.8 ± 5.9
	d=	−1.21	*p* < 0.01	d=	−0.72	*p* < 0.01
Pain score at stretching (mm)	38.4 ± 18.3	12.4 ± 19.7 *	−26 ± 13	30.2 ± 13.9	17.7 ± 15.8 *	−12.47 ± 6.2
	d=	−1.42	*p* < 0.01	d=	−0.87	*p* < 0.01
Knee extension strength (N/kg)	1.2 ± 0.4	1.5 ± 0.5 *	0.36 ± 0.2	1.2 ± 0.5	1.6 ± 0.5 *	0.3 ± 0.2
	d=	0.79	*p* < 0.01	d=	0.63	*p* < 0.01
10-m walking test (s)	14.2 ± 5.5	11.4 ± 3.5 *	−2.8 ± 1.4	14.9 ± 5.0	12.5 ± 2.8 *	−2.4 ± 1.2
	d=	−0.65	*p* < 0.01	d=	−0.64	*p* < 0.01
TUG (s)	17.2 ± 6.0	12.8 ± 3.5 *	−4.5 ± 2.2	20.0 ± 4.6	14.2 ± 3.7 *	−5.9 ± 2.9
	d=	−0.97	*p* < 0.01	d=	−1.47	*p* < 0.01
One-leg standing time (s)	4.9 ± 5.5	6.7 ± 5.1 *	1.7 ± 0.9	4.9 ± 5.8	8.4 ± 8.3 *	3.5 ± 1.7
	d=	0.34	*p* < 0.01	d=	0.51	*p* < 0.01

Data are presented as mean ± standard deviation. Δ Change = Amount of change from PRE to POST. * *p* < 0.05, a significant difference between PRE and POST. ROM, range of motion; TUG, timed up-and-go test.

## Data Availability

All data supporting the conclusions of this study will be fully provided upon request by the authors.
